# GPT-3.5 altruistic advice is sensitive to reciprocal concerns but not to strategic risk

**DOI:** 10.1038/s41598-024-73306-x

**Published:** 2024-09-27

**Authors:** Eva-Madeleine Schmidt, Sara Bonati, Nils Köbis, Ivan Soraperra

**Affiliations:** 1 Center for Humans and Machines, Max Planck Institute for Human Development, Lentzeallee 94, 14195 Berlin, Germany; 2 Research Center Trustworthy Data Science and Security, University Duisburg-Essen, Duisburg, Germany; 3 Max Planck School of Cognition, Leipzig, Germany

**Keywords:** Computer science, Psychology

## Abstract

Pre-trained large language models (LLMs) have garnered significant attention for their ability to generate human-like text and responses across various domains. This study delves into examines the social and strategic behavior of the commonly used LLM GPT-3.5 by investigating its suggestions in well-established behavioral economics paradigms. Specifically, we focus on social preferences, including altruism, reciprocity, and fairness, in the context of two classic economic games: the Dictator Game (DG) and the Ultimatum Game (UG). Our research aims to answer three overarching questions: (1) To what extent do GPT-3.5 suggestions reflect human social preferences? (2) How do socio-demographic features of the advisee and (3) technical parameters of the model influence the suggestions of GPT-3.5? We present detailed empirical evidence from extensive experiments with GPT-3.5, analyzing its responses to various game scenarios while manipulating the demographics of the advisee and the model temperature. Our findings reveal that, in the DG Dictator Game, model suggestions are more altruistic than in humans. We further show that it also picks up on more subtle aspects of human social preferences: fairness and reciprocity. This research contributes to the ongoing exploration of AI-driven systems’ alignment with human behavior and social norms, providing valuable insights into the behavior of pre-trained LLMs and their implications for human-AI interactions. Additionally, our study offers a methodological benchmark for future research examining human-like characteristics and behaviors in language models.

## Introduction

Novel technical architectures such as transformers and extensive text corpora as training data have enabled recent breakthroughs in Natural language processing (NLP). With the advent of ChatGPT, this technological progress quickly became accessible to millions of users. By now, people can easily make use of multiple pre-trained large language models (pre-trained LLMs) to generate suggestions for various everyday activities, ranging from computer coding, automatic translations, and a plethora of writing tasks^1–3^. In view of the growing use of LLMs for everyday advice, concerns arise regarding their response patterns and their consequent impact on human behavior. Underlying these concerns: humans often follow AI advice^4,5^, even if it encourages people to break ethical rules^6^.

The classic approach to understanding the output of computer models has been to take a look “under the hood” of the machine. However, this approach does not work with LLMs. The technical design of such models is often intransparent and highly complex, and therefore, generative AI models produce more unpredictable outputs^7^. One recent approach to gaining a better understanding of the performance of LLMs is observing their behavior in controlled experiments akin to how social scientists observe humans’ behavior^8^. Thus, a growing number of studies have started to systematically probe LLMs’ responses to different prompts^9,10^. For instance, socio-demographic prompting entails systematically sending multiple prompts with minor changes along socio-demographic features in the text^11^.

Such studies have revealed varying degrees of resemblance to human-like behavior across domains such as ethical norms, logical reasoning tasks, personality facets, and moral judgments, highlighting similarities and divergences in LLM behavior compared to human responses^12–14^. For instance, several studies have sought to understand the political colorings of LLMs. One paper indicates that early versions of ChatGPT show a green-left-leaning political bias when prompted to answer questions about politics^15^. More recent studies document racial biases in AI advice^16^. Namely, some LLMs systematically suggest less prestigious jobs when prompts are written in African-American English, and defendants described in this dialect spoken by millions of Americans were more likely to receive a death penalty recommendation by an LLM compared to prompts written in “standard” English^16^. An additional concern arises from research showing that people by no means ignore AI-generated suggestions and advice but are, in fact, often altering their views, beliefs, and behavior based on it^17^.

While AI advice has received attention in political and ethical domains, less is known about more fundamental social tendencies of LLMs. As inherently social beings, humans face countless social situations in their daily lives^18–20^. Frequently, such situations present trade-offs between conflicting goals and norms. For instance, whether one should sacrifice one’s own resources to help others or how much one should trust unknown others. Especially when people’s interests and motivations clash, they seek advice to decide what to do. Here, LLMs increasingly become trusted advisors^1,21^. AI advisors have the advantage of always being available and quick to offer advice for any given query. A systematic investigation of the type of advice LLMs give in such social situations is lacking. Therefore, whether LLMs mimic humans’ notions of other-regarding concerns remains largely unknown.

To gain insights into the advice provided by LLMs for some of the most basic social human behaviors, we draw on economic games. Instead of assessing what people *say they would do,* these economic games allow us to observe what people *actually do* when facing a decision with financial consequences for themselves and others. Therefore, economic games offer a clear and straightforward measure of how people trade off their and others’ well-being in different social situations^22^. Due to this wide popularity of gauging human social behavior using simple decisions, economic games are also becoming an increasingly popular tool for studying machine behavior ^23–27^.

A recent line of research has used systematic prompting to understand LLMs’ social preferences better^28^. One study has used natural language descriptions of altruism and selfishness to introduce human-like behavior of LLMs in economic games^30^. Besides investigating the capacity of GPT-3.5 to manifest human-like social preferences, others have studied the preferences already incorporated in this model (text-davinci-003). Johnson & Obradovich^9^ let GPT-3.5 play a Dictator Game, i.e., a task in which a decision-maker has to divide a financial resource between themselves and a passive recipient. The results show that GPT-3.5 allocates as much money to human partners as humans do, resembling behavior consistent with human altruism. Brookins and DeBacker^10^ study LLM’s behavior in the Dictator Game and prisoner dilemma. The latter describes a canonical task to assess cooperation. They argue that GPT-3.5 (gpt-3.5-turbo) replicates human tendencies towards fairness and cooperation. Compared to human interactions, the model exhibited elevated levels of altruism and cooperation in the Dictator Game and Prisoners’ Dilemma, respectively. Previous studies have similarly found that GPT-4 overestimates altruism when predicting human behavior in the Dictator Game^29,30^.

Our paper adds to this line of research in two main ways. First, we assess the LLM’s strategic and reciprocal tendencies when giving advice by using two economic games: the Dictator Game (DG) and the Ultimatum Game (UG). Both games are two-person games with a decision-maker deciding how to share an amount of money with a recipient. While the DG reflects a non-strategic decision as the recipient is merely passive, the UG adds a strategic component to this social situation. Namely, here, the sender *proposes* a division of the resources that the responder can either accept or reject, leaving both players with nothing. Therefore, we introduce the novel approach to compare LLM’s behavior across two structurally similar but strategically distinctive games, allowing us to disentangle the model’s sensitivity for the fairness of the outcomes and intentions. This way, we gain novel insights into whether LLMs social preferences are sensitive to intentions. It is important to note that we are not measuring how the LLM itself would behave, but rather its suggestions and advice on how to behave in these scenarios.

Second, we manipulate the prompt and the technical details of the LLM to assess the robustness of the advice. On the one hand, we compare the LLM’s suggestions for different demographics by manipulating the age and gender of the person receiving the advice. This approach allows us to explore whether suggestions are homogenous or different people would receive different advice. On the other hand, we manipulate the technical parameter of LLM’s temperature. This parameter is crucial in determining the randomness and creativity of the LLM’s output. A lower temperature leads to more deterministic and potentially repetitive responses, as the model prefers the most likely following words. Conversely, a higher temperature encourages the model to explore less likely options, injecting diversity and creativity into the responses. The reason to include the temperature manipulation is twofold: first, this serves as a robustness check regarding the stability of model responses to an increase in the noise of the token selected; second, since manipulations of temperature have been associated with “personality changes” of the model^14^, it allows to explore the impact of heterogeneity in personality and creativity.

While a rich collection of behavioral studies has documented how humans behave in economic games, research on LLMs’ behavior in such tasks is in its infancy. Many behavioral studies have examined how people behave in the canonical games DG and the UG, allowing the aggregation of empirical insights in large meta-analyses (see meta-analyses on DG^31^ and UG^32^). A diverse overview exists of how demographic factors, such as age^31,33^ and gender, shape behavior in economic games (DG^31^; UG^34–36^). Likewise no clear relationship between prompted gender and behavior in LLMs appears to exist—at least in terms of responses to the personality inventory^14^. However, evidence on how socio-demographic prompting of these features influences LLMs’ advice remains scarce.

Our work differs from prior studies by comparing LLM behavior in two distinct economic games to assess sensitivity to fairness and intentions. Unlike prior studies, we further examine GPT-3.5’s advice versus typical human behavior by manipulating demographic prompts and technical parameters to evaluate robustness. This approach provides a nuanced understanding of GPT-3.5’s social preferences and alignment with human decision-making.

### Research questions and identification strategy

Specifically, we aimed to answer three main research questions that we pre-registered on As.predicted (see https://aspredicted.org/RXX_NM8).

The first question (Q1) assesses whether GPT-3.5 suggestions are sensitive to strategic considerations, thus following a human-like pattern, or are purely motivated by altruism. To test this notion of strategic sensitivity, we prompted GPT-3.5 to advise senders in the DG and UG on how much of their initial endowment they should send to a recipient (see more details in Methods). To test whether the LLM suggestions are sensitive to the risk of being rejected, we can compare the average amount suggested by the LLM in the UG and in the DG, where the risk is absent. If the amounts suggested in the UG significantly exceed those in the DG, the LLM reflects this strategic consideration in its advice.

The second question (Q2) examines whether GPT-3.5 suggestions incorporate the concept of positive reciprocity, i.e., if GPT suggestions reward a previous kind action of the counterpart. To investigate whether the model suggests a generous response to a kind action, we prompted the GPT-3.5 with a scenario describing a previous interaction in which the receiver in the DG made a monetary gift to the sender. If suggestions are sensitive to positive reciprocity, the amounts suggested should be increasing in the size of the gift, and eventually higher than in the standard version of the DG where the kind action is not present.

The third question (Q3) tests whether GPT-3.5’s suggestions incorporate costly punishment of bad intentions. Specifically, we explore the influence of negative reciprocity on the suggestion to reject unfair offers by studying whether suggestions to reject stem from the unfairness of the outcomes alone or involve a retaliatory response to unfair behavior. In other words, we aim to determine if the advice to reject unfair offers is based solely on the unfairness of the offer or if it also includes a response to get back at the person’s unfair behavior. To do so, we compare the rejection suggestions made to a responder facing an unfair offer in the UG—for instance, receiving only 10% of the total amount while the sender retains 90%—with the suggestions given to an individual presented with an equivalent binary choice outside the UG context—i.e., the suggestion of what to choose between the option of keeping 10% of an amount of money for yourself and giving 90% to another person and the option of both players obtaining nothing. This design allows us to keep the (un)fairness of the outcomes constant while manipulating the way such (un)fairness originates. If suggestions are sensitive to outcome fairness, we expect to observe rejection suggestions to increase with the inequality of the final distribution. Moreover, if suggestions are driven by negative reciprocity, we expect to observe more suggestions to reject when the decision follows from an unfair proposal.

## Methods

### Technical details

We used Python (version 3.10.8) to query the pre-trained OpenAI text-davinci-003 LLM (GPT-3.5) through the OpenAI API and to perform data pre-processing. We conducted the data analysis and visualization in R (version 4.3.1).

## Design

### The tasks

To answer our research question, we employ the two games mentioned above: the Dictator Game (DG) and the Ultimatum Game (UG). The DG is a two-person game where a person (the dictator) is given a certain amount of money and must decide how to split it between themselves and the other person (the recipient). The recipient has no influence on the decision and must accept whatever amount the dictator chooses to give. The DG is often used to study altruism and reciprocity^27,31,37^.

The UG is also a two-person game where a person (the proposer) is given a sum of money and proposes a division between themselves and a second person (the responder). The responder then decides whether to accept or reject the offer. If the responder accepts, both players receive the proposed amounts. If the responder rejects the offer, both players receive nothing. The UG is often used to study strategic negotiation and fairness considerations^38–40^. In this paper, we use the following variations of the games:

- **Dictator Game (DG):** The dictator is given 10 Euros and can divide this money as they please between themselves and the recipient, who is passive and cannot make any decision.
- **Dictator Game with reciprocity (DG-R):** The same as the DG, with the only difference that previous to the current interaction, the recipient gifted the dictator an amount of money that can take the following values: 1, 2, 3, 4, 5, and 10 euros.
- **Ultimatum Game (UG):** The proposer is given 10 Euros and can propose a division of this money between themselves and the responder. In this case, the responder can choose whether to accept the proposal, obtaining the proposed share for themselves and leaving the rest of the money to the proposer, or to reject the proposal, leaving both of them with nothing.
- **Binary Dictator Game (B-DG):** A variation of the previously described DG, where the choice of the dictator is restricted to two possible options: zero euros for both players or a specific split of the 10 euros. This game serves as the benchmark comparison for the responder’s rejection rates in the UG.

### Prompts of the games

We designed game-specific prompts closely resembling human participant study descriptions to ensure comparability. GPT-3.5 was not portrayed as an independent agent nor incentivized to provide specific advice, e.g., such as being generous; instead, prompts were used to seek model-generated suggestions for player behavior. This choice stemmed from two considerations: (a) the model cannot answer for itself, as its training data does not encompass knowledge about the qualities of GPT-3.5, a limitation previously highlighted^41^, and (b) asking for behavioral suggestions aligns with potential real-world use cases that could affect human behavior, as discussed above. We provide the exact prompts used in this study in the supplementary material (see supplementary material, Material B). No human participants were involved in this study. To ensure that recommendations are based on a formally correct understanding of the games, allowing for a meaningful interpretation by humans, we asked the model to answer comprehension questions similar to those typically used for human participants in behavioral experiments (see supplementary material, Material C).

We queried GPT-3.5 using prompts describing these tasks from the perspective of one of the players who is asking for suggestions on how to behave and asked to provide answers in a structured json format (see supplementary material, Material B). Specifically, we submitted prompts where:

1. Dictators in the DG and proposers in the UG ask for suggestions about the amount X to send to the other player (X from 0 to 10);
2. Responders in the UG ask for suggestions on whether to accept or reject an offer of Y euros (with Y in {1, 2, 3, 4, 5, 10});
3. Dictators in the DG with reciprocity ask for suggestions about the amount X to send to the recipient (X from 0 to 10) given that the recipient gifted them an amount Y before the interaction (with Y in {1, 2, 3, 4, 5, 10});
4. Dictators in the binary DG ask for suggestions on what to choose between the option to keep Y euros for themselves and give 10—Y euros to the recipient (with Y in {1, 2, 3, 4, 5, 10}) and the option of both players obtaining zero euros.

**Fig. 1:**
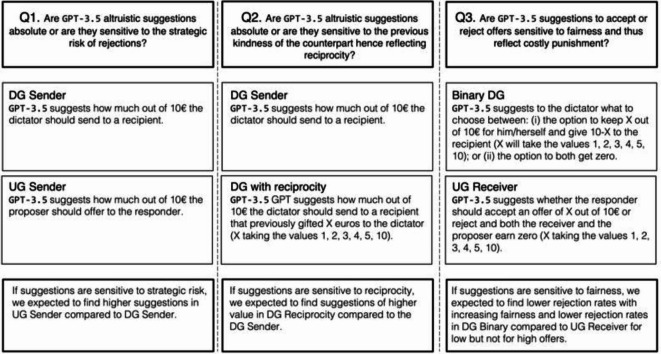
Subordinated Questions to Research Question 1 (To what extent do GPT-3.5 suggestions reflect human social preferences?) and Experimental Design. *Note*. Q1 = Question 1, Q2 = Question 2, Q3 = Question 3, DG = Dictator Game, UG = Ultimatum Game.

In Fig. 1, we illustrate how the replies to these prompts map onto and allow us to answer each research question. The supplementary material presents a more detailed overview of the experimental design (see supplementary material, Material A).

### Prompt manipulations

To assess robustness of the advices, we systematically manipulated the temperature of the language model, which is related to the creativity of the replies, and the demographic features of the person asking for suggestions. As for the temperature, we considered three levels: below default (temperature = 0.5); OpenAI default (temperature = 1); and above default (temperature = 1.5). As for the features of the person asking for advice, we included demographic information to some versions of the prompt. We systematically manipulated the demographics along two factors: the age of the person receiving the suggestion (with three levels: 18–30 yrs, 31–50 yrs, and 51–70 yrs) and the gender of the person receiving the suggestion (with three levels: female, male, and non-binary). For each prompt, we collected 1000 replies for each temperature level without including demographic information (*unprompted demographics*) and 1000 replies for each temperature level and combination of the demographic factors (see supplementary material, Material D for a more detailed breakdown of the variations).

In the pre-registration, we planned to collect 10 responses for each prompt variation using a temperature of 0 to assess the model responses when LLM predictions are deterministic. We failed to collect such responses due to a coding mistake, and by the time we realized that such data was missing, OpenAI had already updated the model. We therefore decided not to collect such information.

## Analysis and results

The pre-processing steps are detailed in the supplementary material (Material E). The pre-registration of the analyses can be found under the following link: https://aspredicted.org/RXX_NM8. For all the questions, we performed separate tests for each temperature level and a single test collapsing the temperature. The main analysis collapsed the answers for the different levels of demographics. To additionally test whether and how age and gender influence the responses of the model we plotted the dependent variables grouped by age and gender for each question and conducting subsequent regression analyses.

The analysis included 599,244 valid responses out of 600,000 prompts submitted (see supplementary material, Material F for an overview of the number of observations per game and temperature). Invalid responses are the ones for which the model either provided a value in the json response that did not comply with the type requested or it did not provide a value in the json response and it was not possible to infer the value from the text (see supplementary material, Material E for the details).

### Q1: Are GPT altruistic suggestions sensitive to strategic considerations?

**Fig. 2:**
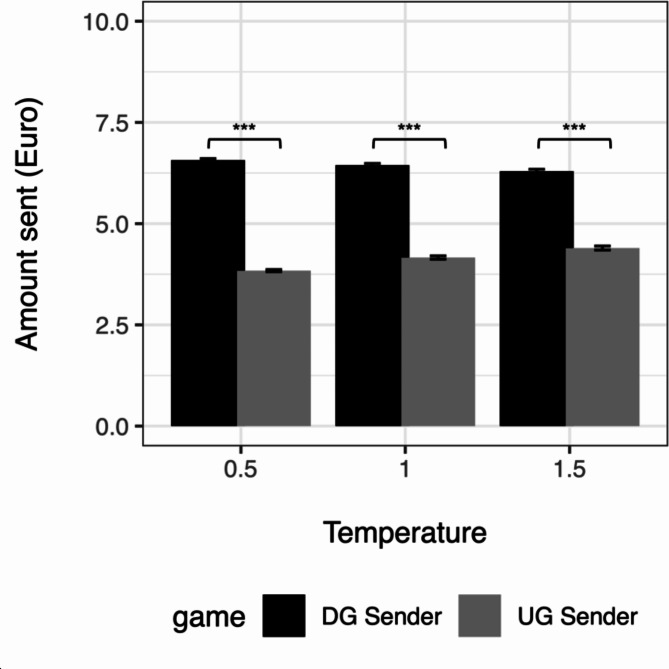
Average suggested amount sent in DG Sender and UG Sender by Temperature Level. *Note.* Average suggestions of GPT-3.5 in the Dictator Game (DG) were higher than those in the Ultimatum Game (UG). The *p*-values associated with each comparison are indicated as follows: *p < 0.05, **p < 0.01, ***p < 0.001.

To answer (Q1) whether altruistic suggestions are sensitive to strategic considerations or purely driven by altruism, we compared the suggested amount sent in the DG and the suggested amount sent in the UG. Figure 2 shows the average suggestion in the two cases separated by temperature level.

When pooling responses across the different levels of the demographic and temperature factors, we observe considerably more altruistic suggestions in DG Sender (*M* = 6.44, *SD* = 1.57) than in UG Sender (*M* = 4.13, *SD* = 1.55), *t*(59,973) = 180.77, *p* < 0.001, *Cohen’s d* = 1.47 (95% CI [1.46; 1.49]) and results are consistent across different temperature levels (see Table 1). This result indicates that the model’s suggestions do not reflect human behavior when a risk of rejection exists. Contrary to typical results in the behavioral science literature, we observe substantially lower suggested amounts to send when the risk is present than when the risk is not present^42^. Specifically, the amounts suggested by the LLM in the DG are substantially more generous than those of human participants. The amounts suggested by the LLM in the UG are slightly lower than in behavioral experiments with human participants.

**Table 1:** Results of Welch two sample t-test.

	DG sender		UG sender			*p*	*CI*	
	*M*	*SD*	*M*	*SD*			Lower	Upper
All	6.44	1.57	4.13	1.55	*t*(59,973) = 180.77	< 0.001	2.28	2.33
Temp = 0.5	6.57	1.38	3.84	0.99	*t*(*18,169*) = 160.89	< 0.001	2.70	2.76
Temp = 1.0	6.45	1.52	4.16	1.58	*t*(19,962) = 104.32	< 0.001	2.24	2.33
Temp = 1.5	6.30	1.77	4.40	1.90	*t*(19,885) = 73.01	< 0.001	1.85	1.95

*Note.* Suggestions of GPT-3.5 in the Dictator Game (DG) were higher than those in the Ultimatum Game (UG) across all temperature levels. When compared to human behavior, we find more altruistic suggestions in the DG, suggesting that the model’s recommendations do not align with human behavior when there is a risk of rejection.

When looking at the effect of the demographic features of the person receiving the advice, we find robust effects across age and gender. In all cases, the model suggestions are less generous when risk is not present (see supplementary material, Material G). The effect size is also comparable across demographics, except for older advisees, i.e., age 51–70, who receive more extreme advice than the other age groups.

### Q2: Do GPT suggestions incorporate positive reciprocity?

**Fig. 3:**
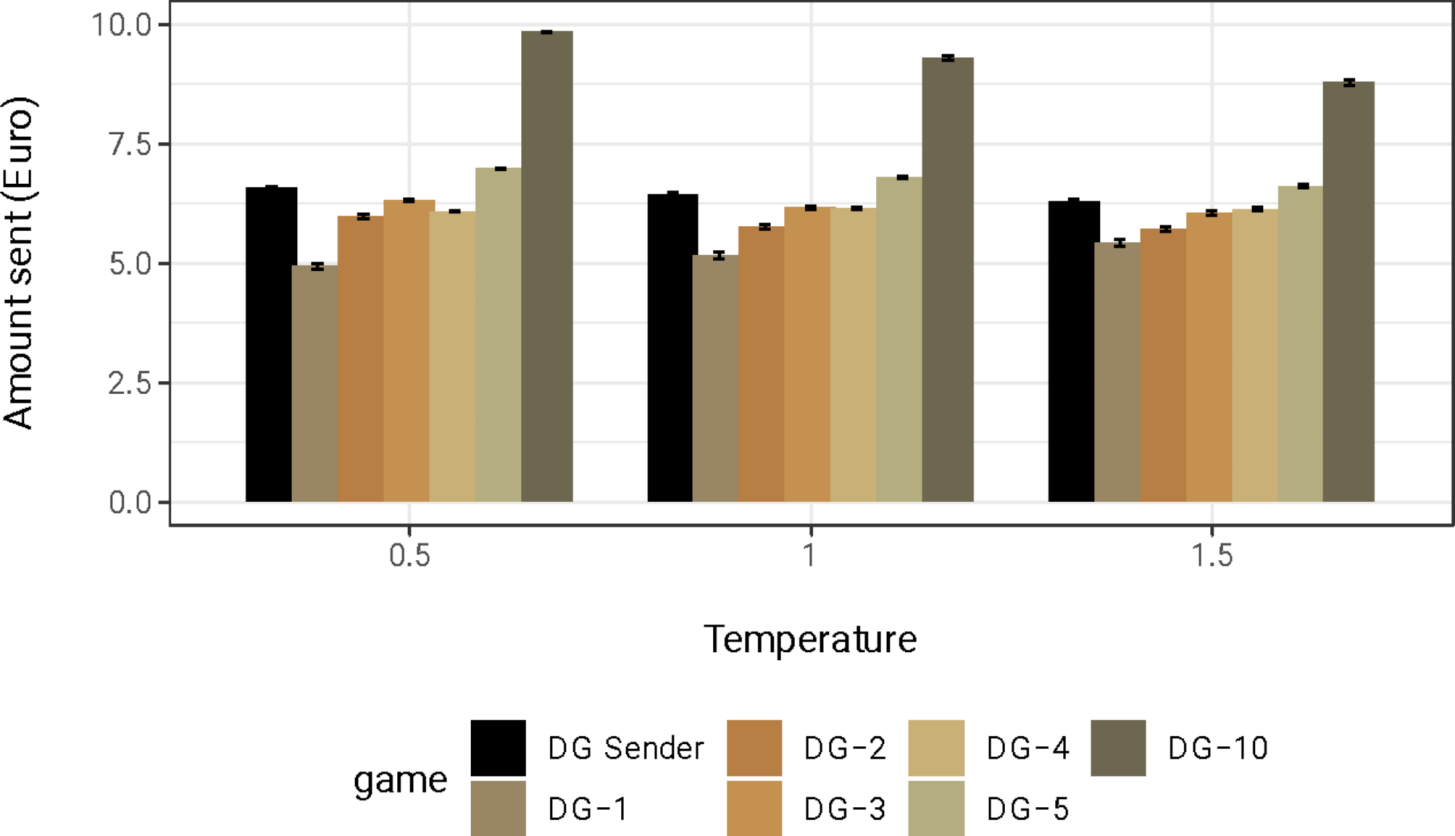
Mean suggested Amount to send in DG Sender and DG Reciprocity by Temperature. *Note*. Suggestions increase with the kindness of the other player’s action (DG-1 to DG-10). Suggestions in the presence of a kind action are more generous than in the absence of a kind action (DG Sender) only when kindness is substantial (DG-10). DG = Dictator Game, DG-*n* refer to DG Reciprocity with *n* depicting the amount of money a receiver previously gifted to the dictator.

To answer (Q2) whether the model’s suggestions reward the kind actions of the counterpart, we look at the suggested amount in the DG Reciprocity conditions. We assess reciprocity in two ways: first, we look at whether suggestions are increasing in the size of the gift received from the counterpart, and second, we compare these suggestions to the suggested amount in the DG Sender, where the kind action is absent. Figure 3 shows the average amount sent in the DG Sender and the DG Reciprocity for the different kindness levels, i.e., 1, 2, 3, 4, 5, and 10 euros. The figure shows that (i) suggestions are indeed increasing with the kindness of the other player’s action, and (ii) suggestions in the presence of a kind action are more generous than in the absence of a kind action only when kindness is substantial.

Linear regressions with the suggested amount as the dependent variable and with both the kindness of the action and a dummy variable capturing the DG Sender as the explanatory variables statistically support these observations (see Table 2). The dummy measures the predicted difference in generosity between the DG Sender and the DG Reciprocity when kindness is zero, comparing suggestions to send money to someone who did not perform a kind act. The estimated parameter for the “Kindness” variable is positive, indicating that suggestions increase with the amount received from the counterpart. Moreover, the estimated parameter for the variable “DG Sender” is significantly greater than zero, indicating that suggestions are higher when kindness is absent compared to when kindness is present. Overall, the results support the existence of positive reciprocity in the LLM’s suggestions as suggested generosity is increasing with the kindness of the other participant. However, we find ambiguous results when comparing these suggestions to those without a generous action. As the figure and the regressions show, these results are robust across temperature levels of the model. When looking at robustness across demographic, we observe consistent patterns for all the demographic features (see supplementary material, Material H).

**Table 2:** OLS regression with amount sent as the dependent variable.

	All	Temp = 0.5	Temp = 1.0	Temp = 1.5
Kindness	0.448*** (0.001)	0.517*** (0.002)	0.450*** (0.002)	0.376*** (0.003)
DG Sender	1.734*** (0.012)	2.031*** (0.016)	1.763*** (0.020)	1.407*** (0.025)
Prompted demographics	− 0.157*** (0.015)	0.049* (0.020)	− 0.219*** (0.025)	− 0.302*** (0.030)
Age (31–50)	− 0.175*** (0.010)	− 0.224*** (0.013)	− 0.161*** (0.016)	− 0.139*** (0.020)
Age (51–70)	− 0.210*** (0.010)	− 0.258*** (0.013)	− 0.225*** (0.016)	− 0.147*** (0.020)
Male	0.257*** (0.010)	0.165*** (0.013)	0.312*** (0.016)	0.293*** (0.020)
Non-binary	0.194*** (0.010)	0.242*** (0.013)	0.203*** (0.016)	0.138*** (0.020)
Constant	4.826*** (0.013)	4.517*** (0.018)	4.842*** (0.022)	5.118*** (0.027)
Observations	209,879	70,000	69,996	69,883
R^2^	0.336	0.523	0.350	0.205
Adjusted R^2^	0.336	0.523	0.350	0.205

*Note.* In DG with reciprocity, suggestions increase with the amount received from the counterpart. Suggestions are higher when kindness is absent (DG Sender) compared to when kindness is present. The results support the existence of positive reciprocity in the LLM’s suggestions, but we find contradictory results when comparing these suggestions to those without a generous action. DG Sender = dummy variable capturing suggestions in the Dictator Game, Kindness = continuous variables capturing the kindness of the other participant action. *p < 0.05; **p < 0.01; ***p < 0.001.

### Q3: Do GPT suggestions incorporate costly punishment for unfair actions?

**Fig. 4:**
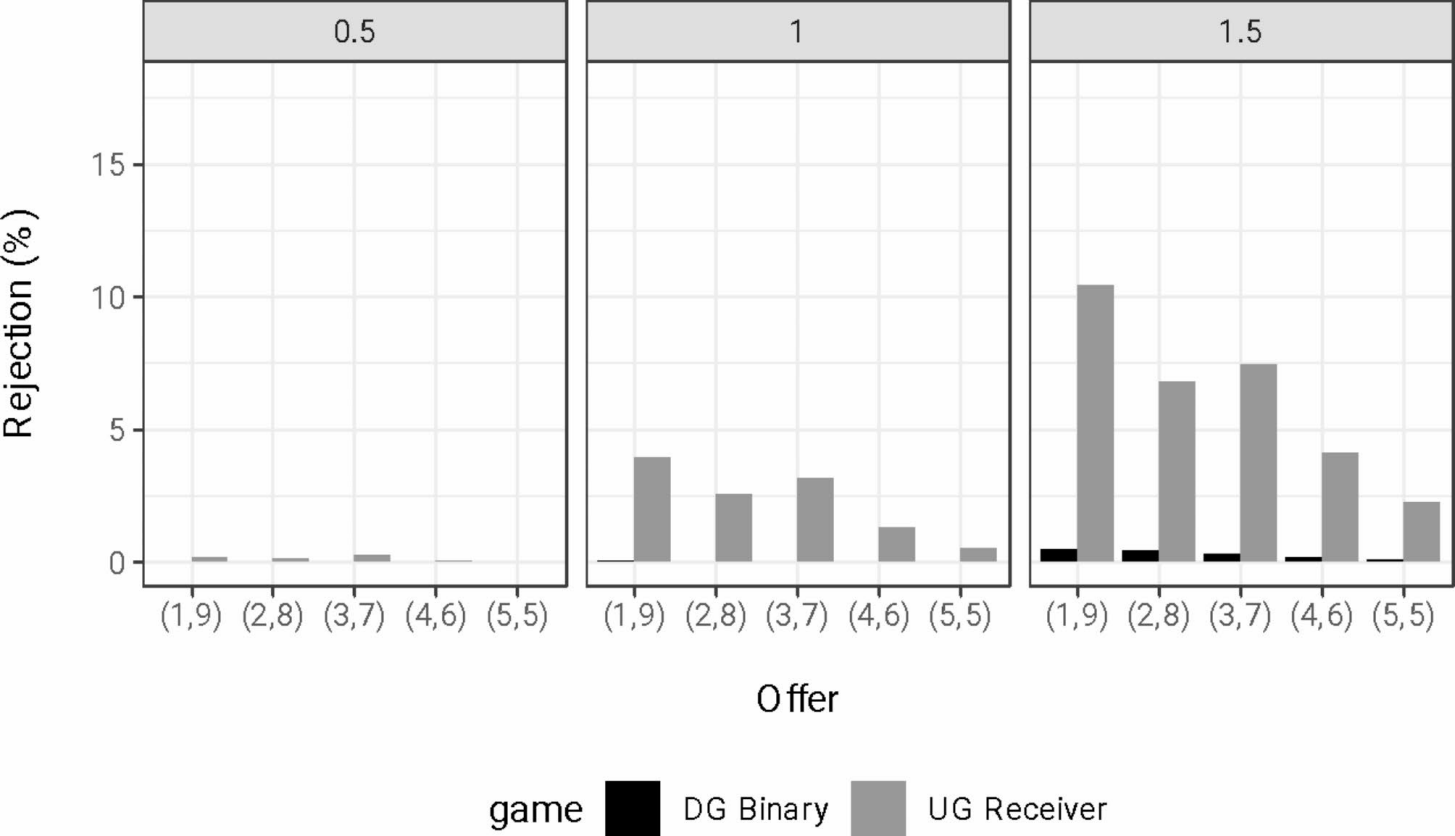
Rejection Rates in DG Binary and UG Receiver per Temperature. *Note.* DG = Dictator game, UG = Ultimatum game. The ordered pairs of values on the x-axis indicate the split of the 10 euros, with the first value representing the amount for the dictator/proposer and the second value representing the amount for the recipient/responder.

To assess the influence of negative reciprocity on the suggestion to reject unfair offers (Q3), we compare GPT suggestions in UG Reciever and in the DG Binary. Figure 4 shows the rate of rejection suggestion for different allocations in the two cases, split by the temperature of the model. The figure highlights various patterns. First, suggestions to reject unfair allocations in binary DG are virtually non-existent for all temperature levels. This result implies that GPT suggestions are not averse to outcome inequality, even when inequality is substantial (1 euro for the decision maker vs 9 euros for the other player).

Second, suggestions to reject unfair proposals in UG strongly correlate with the model’s temperature. Similar to binary DG, suggestions to reject are virtually non-existent at low temperature levels. For higher temperature levels, however, the model starts to suggest rejections of unfair outcomes proposed by the other player. Third, such suggestions are weakly increasing with the level of unfairness. Overall, these results indicate that “cold” GPT models (low temperature) make very rational and self-interested suggestions. Conversely, “warmer” models (high temperature) suggest retaliatory punishment for unfair actions. While these patterns align with human emotional reactions to intentions, rejection rates seldom exceed 10%. This result is in notable contrast with human behavior, where, on average, approximately 16% of UG offers are rejected^39^.

These observations receive statistical support from the regression results in Table 3. The table reports the results of linear probability models with a dummy variable indicating the rejection, i.e., the choice of the option that gives both agents zero euros, as the dependent variable. We included the variable “Fairness”, capturing the amount for the decision maker; the indicator variable “UG Receiver”, capturing choices made in the Ultimatum Game context; and their interaction, as explanatory variables. Estimated parameters in the Table are scaled to show the effects in percentage points. Regression model (2; temperature = 0.5) shows that rejections are virtually non-existent and do not change substantially with the fairness of the outcome and with the intention—e.g., UG Receiver increases rejection suggestions of the most unfair offer by only 0.2 percentage points. Models (3; temperature = 1) and (4; temperature = 1.5) show substantially higher suggestions to reject when unfairness is generated by another person’s decision (the likelihood of rejection of the most unfair offer is 3.95 and 10.56 percentage points higher in the context of the UG for the medium and high temperature, respectively) and a strong relationship between rejection suggestions and the fairness of the allocation in the UG context (Compared to the DG context, for the medium and high-temperature suggestions to reject in the UG decrease by 0.99 and 1.98 percentage point for each extra euro allocated to the decision-maker). When looking at these patterns across different demographic features of the person receiving the suggestion, we find qualitatively robust results (see supplementary material, Material I). At the same time, some differences in the intensity of negative reciprocity emerge, with rejection suggestions decreasing with age and slightly higher for the non-binary gender category.

**Table 3:** OLS regressions with rejection rates as the dependent variable.

	All	Temp = 0.5	Temp = 1.0	Temp = 1.5
Fairness	0.039*** (0.006)	0.000 (0.0001)	0.010* (0.005)	0.107*** (0.018)
UG—Receiver	4.904*** (0.090)	0.214*** (0.031)	3.954*** (0.139)	10.560*** (0.224)
Fairness × UG—Receiver	− 1.010*** (0.098)	− 0.078* (0.039)	− 0.988*** (0.154)	− 1.980*** (0.245)
Prompted demographics	− 0.090 (0.057)	0.000 (0.021)	− 0.097 (0.087)	− 0.175 (0.142)
Age (31–50)	− 0.313*** (0.055)	− 0.027 (0.019)	− 0.307*** (0.083)	− 0.610*** (0.139)
Age (51–70)	0.209*** (0.051)	0.010 (0.016)	0.217*** (0.076)	0.399*** (0.131)
Male	0.661*** (0.056)	0.063** (0.021)	0.671*** (0.085)	1.252*** (0.141)
Non-binary	− 0.954*** (0.031)	− 0.043*** (0.010)	− 0.816*** (0.046)	− 2.005*** (0.077)
Constant	0.585*** (0.082)	0.056 (0.032)	0.713*** (0.127)	0.993*** (0.205)
Observations	299,578	100,000	99,949	99,629
R^2^	0.022	0.001	0.019	0.047
Adjusted R^2^	0.022	0.001	0.019	0.047

*Note.* Rejection likelihood increases significantly with unfair offers and varies with temperature settings. UG – Receiver = dummy variable capturing suggestions to the receiver in the Ultimatum Game, Fairness = amount of money to the receiver; *p < 0.05; **p < 0.01; ***p < 0.001.

We further repeated the regression using logit and probit models (see supplementary material, Material J). In contrast to the OLS regression, these show that fairness increases the likelihood of rejection and that UG Receiver variable decreases rejection likelihood. This indicates that the relationship between fairness, the UG context, and rejection behavior is complex and that results should be interpreted with caution.

### Further data exploration and answers to the validation checks

In the supplementary material (Material K), we provide the analysis of the GPT’s suggestions regarding rejections of very generous offers, i.e., offers in which the proposer sends the whole 10€ to the responder. We included these prompts as a stress test for situations that rarely occur in human interactions. Suggestions in DG Binary showed strong tendencies towards inequality aversion, with suggestions to reject in more than 35% of the cases. This result starkly contrasts with the virtually non-existent aversion to inequality observed for unfair offers. Moreover, suggestions to reject in UG Receiver, where intentions are present, are rarely made—a result that is consistent with humans rarely rejecting generous offers.

### Control questions

As for the answers to the control questions, these can help interpret the coherence of GPTs’ suggestions. Starting with the model answers to the control questions for the DG, the model consistently yielded accurate responses across all ten instances of prompting. In the case of UG Sender, however, invalid responses were observed for the question: "If person A proposes to send 2 Euros to person B and person B rejects, how much money does person A have in the end?". Here, the model consistently answered 10€ instead of 0€. This response suggests that the model has trouble correctly dealing with the more complicated strategic setting. Interestingly, the answer would have been accurate within the DG context, suggesting that the model suggestions may not fully differentiate the two contexts.

Within the UG Receiver, we observed several inconsistencies. Specifically, in response to the question: "If person A proposes to send 2 Euros to person B and person B accepts how much money does person B have in the end?", the model provided the incorrect answer of 7€ eight times and the correct answer of 2€ only twice. Additionally, for the question: "If person A proposes to send 2 Euros to person B and person B rejects how much money does person B have in the end?”, the response was incorrect in all instances. Also, the model does not correctly deal with prompts entailing strategic interactions in this case. Analyzing GPT’s suggestions in these cases is still informative because, in real-world scenarios, people seeking advice are unlikely to notice these inconsistencies, as the responses to advice-seeking will probably not reveal them.

## Discussion

This study focuses on understanding the fine-grained aspects of social preferences in GPT-3.5 suggestions. Our study builds on existing research by investigating GPT-3.5’s alignment with human-like social preferences by testing them using well-established paradigms for social behavior. We extend previous work beyond altruism by examining the nuances of reciprocity and costly punishment. Moreover, we systematically analyzed the influence of demographic and technical parameters with the aim of discerning the extent to which GPT-3.5 suggestions reflect human social preferences and to identify factors that shape its responses.

As the first main research question we assessed whether GPT-3.5’s suggestions reflect strategic considerations related to the risk of rejection, comparing the offers in the DG to those in the UG. Surprisingly, we observed that DG offers exceeded UG offers. This finding contradicts the typical pattern observed in human participants, wherein UG offers tend to be generally higher, driven by the inherent risk of rejection and the associated potential for a 0–0 outcome^42–44^. This finding is largely consistent across gender and age of the advisee—with deviations only for advisees aged 51–70 years. Hence, LLM suggestions substantially deviate from typical human behavior in these different social settings. Namely, suggested mean offers in the DG were, on average, much higher (64.4%) than previously observed human behavior (~ 30%)^31^. This difference underscores the model’s notably generous and seemingly altruistic disposition compared to human decision-making. These results do not align with previous findings documenting that model suggestions largely resembled humans’ sharing rates in the DG^9^. Instead, they confirm results by Brookins & DeBacker^10^, showing that UG offers aligned more closely with typical human behavior (4€ in UG^39^; in model = 4.13).

GPT-3.5’s suggestions demonstrate a level of generosity that surpasses typical human behavior. Speculatively, there could be a disparity between our written advocacy for the importance of altruism and our actual behavior (we do not *practice what we preach*). This tendency towards generosity in the training data could influence the model’s predisposition towards more altruistic suggestions the DG. It could further be speculated that an altruistic tendency was deliberately introduced by OpenAI, for example, in the context of reinforcement learning from human feedback, to render the model generally more amicable and prosocial in its suggestions. This is in line with recent work indicating that the newer version of GPT-3.5 (GPT-4) prioritizes the well-being of others over individual well-being (using a similar methodological approach to ours^45^). This does not, however, explain why UG offers resemble typical human behavior. One potential reason for GPT-3.5’s behavior in the binary ultimatum game could be its inherent simplicity and binary nature, aligning more closely with the model’s training data. Alternatively, the model might respond better to explicit fairness cues in this game. The varying complexity and context of different ultimatum game scenarios may also influence the model’s responses, causing the observed inconsistencies. Additionally, the disparity between the LLM’s and human offers in the DG contradicts the assumption that research of this nature may inadvertently yield more insights into the survey responses of the reference population rather than unveiling the inherent characteristics of the language models themselves^46^.

As a second main question, besides LLMs capturing general notions of strategic risks, our study explored its sensitivity to reciprocity. Therefore, we compared the suggested offers in a DG following a benevolent action of the recipient to the suggested offers in the standard DG Sender, where a benevolent action towards the dictators before the game was absent. Overall, we find mixed evidence of positive reciprocity in the model suggestions. We find that suggested offers increase with the niceness of the other participant’s gesture, supporting that GPT-3.5’s suggestions reflect human-like reciprocity^47,48^. However, the fact that in the absence of a generous action (DG Sender), suggestions are more generous than for low levels of niceness (DG Reciprocity) raises some doubts that warrant further consideration regarding the interpretation of these results.

The disparities in responses between DG with reciprocity and DG Sender may be attributed to contextual differences. In DG with reciprocity, where the recipient’s action influences the suggestions, the model demonstrates sensitivity to the perceived generosity of the other participant. In contrast, in DG Sender, which lacks knowledge about the other participant, the LLM indicates a general predisposition towards high altruism, as commented above. This suggests that the model’s behavior may vary based on the contextual information available. Notably, the observed pattern aligns with human behavior in sequential DGs. It is, thus, possible that GPT-3.5 interpreted the prompt as a sequential DG. It is further possible that GPT-3.5 treats the DG as a social interaction emphasizing kindness and altruism, while it views the UG as competitive. However, this remains speculative, as our design does not allow us to measure such social interpretations of LLMs.

As a third question, we assessed whether the model’s suggestions capture the costly punishment of unfair offers. We did so by comparing two settings: rejections of unfair outcomes when these are the results of the action of another person (UG Receiver) versus when they are simply allocations one can choose from (Binary DG). Overall, our results indicate that LLM’s suggestions to reject reflect the unfairness of the action. Rejection rates increase with the relative unfairness in the UG Receiver setting. Interestingly, we find no support for inequity aversion as rejection rates remained close to zero in all conditions in the binary DG setting.

Our findings generally reveal that rejection suggestions occur less frequently than in human behavior. Namely, rejection rates reached a maximum of 10%, starkly contrasting the 40% rejection rates observed in human participants in the UG^39^. Moreover, LLM’s suggestions to reject decreases with lower temperature levels. This novel result suggests that lower temperature levels lead to decisions commonly considered “rational” in a game-theoretic sense, with suggestions aligned with the maximization of one own monetary payoff. We find a resemblance between the dependence of rejection rates on the perceived unfairness of offers in GPT-3.5 suggestions and the expected patterns exhibited by human counterparts^49^.

### Methodological considerations

The novel approach to letting the LLM complete comprehension check items provides another indication of LLM inconsistencies. The results revealed that the model exhibited limitations in grasping more intricate implications within the UG. However, understanding such nuances may not be a prerequisite for real-life applications. Our analysis indicates that the model’s responses remain consistent with what we would expect from a human-generated answer, highlighting the potential implications of model behavior on human behavior, irrespective of an internal understanding of the scenarios on the side of the LLM. GPT-3.5’s recognition of DG and UG paradigms may influence its recommendations more than model’s inherent values or game context. Our study did not include a probing the reasoning behind GPT’s suggestions, which, on the one hand, would have provided deeper insights into the underlying mechanisms but, on the other hand, would have been impractical due to computational constraints and the need to compare suggestions with results in human research. We tried to tap into the model’s understanding of the game by using comprehension questions, which are the standard used in human research. This study further relies on statistical tests of GPT-3.5’s responses. Besides temperature, we did not examine other specific LLM components that generate these responses and influence strategic behavior study.

On a technical note, while the relationship between temperature and rejection rates can be given an emotional interpretation, with suggestions at lower temperatures being cold (i.e., not emotional) and rationally self-interested and suggestions at higher temperatures being war (i.e., emotional) there could be a mechanical explanation for this pattern. Since higher temperatures make the output of the model noisier, in cases where the model is forced to provide only one out of two tokens and the confidence is low enough, one can observe that the rate of the two tokens gets closer to 50% when increasing the temperature. Still, while a technical explanation for the effect can be provided, human recipients of the advice may ascribe emotionality to the underlying motives of the suggestion.

On a methodological note, we believe that our research provides a foundation for future investigations and offers a benchmark for methodological approaches examining human-like characteristics and behaviors in LLMs. As AI systems continue to play an increasingly significant role in various applications, comprehending their behavior and the factors that influence it becomes paramount to ensure responsible and ethical AI interactions. Since we have no access to the model and training itself, we need to rely on mapping the responses to standardized prompts to understand the model’s social preferences. By not granting such access AI companies prevent researchers from understanding to what extent the training data itself, the model or later steps such as reinforcement learning with human feedback^50^ are responsible for the outputs.

Note that our study compares GPT-3.5 advice to human behavior in studies where they are not advisors but make decisions for themselves. As we argue in the discussion, the observed differences may be the results of a discrepancy between our written advocacy for the importance of altruism and our actual behavior. Additional data comparing human and machine suggestions is required to better understand this disparity. Existing research has already explored the question whether people follow such AI-generated versus human-written advice to a similar extend. These results show that the source of the advice (AI vs. human) matters less than whether the advice aligns with people’s self-interest as people follow both types of advice self-serving ways^6^. Alternatively, one could explore the LLM’s behavior as an agent in these economic games and compare human behavior to machine behavior. Future studies could explore eliciting GPT-3.5’s direct behavior to further understand its social preferences compared to the current advice-based approach.

## Conclusion

So, are LLMs good advisors in strategic situations? The answer, as so often, is it depends. For some settings, GPT-3.5 captures nuances of social situations. Overall, we find mixed results regarding the internal consistency of GPT-3.5 suggestions, which in some cases align with well-known behavioral patterns observed in human subjects and in others don’t. On the one hand, we find a remarkable degree of internal consistency within single decision settings, with altruism increasing in the niceness of the counterpart’s action and with rejection rates in UG being dependent on the fairness of the offer. On the other hand, we document inconsistencies across decision settings, with offers in the presence of risk of rejection being lower than offers without risk of rejection. This result raises a warning regarding the use of single tasks to probe LLMs and suggests adopting a multi-task approach, which permits a better assessment of consistency. Our finding that GPT-3-5 does not capture the strategic differences between both settings adds to the (growing) list of contexts where LLMs seem to fall short of human-like performance. Hence, blindly following suggestions by the LLM in strategic situations can also backfire. This study suggests that LLMs may not always behave as expected from a human agent in strategic decision-making scenarios and emphasizes caution when considering relying on advice provided by these models.

## Supplementary Information

Supplementary Information.

## Data Availability

https://osf.io/shq8x/?view_only=52b2aa6871384fc4978dc1ec3100b575.

## Code availability

https://osf.io/shq8x/?view_only=52b2aa6871384fc4978dc1ec3100b575.
