# Supplementary material for: GPT-3.5 altruistic advice is sensitive to reciprocal concerns but not to strategic risk
Source: Sci Rep. 2024 Sep 27;14:22274. doi: 10.1038/s41598-024-73306-x (PMC11436787; doi:10.1038/s41598-024-73306-x)
Supplement: Supplementary file 1 — Supplementary Information. [file 41598_2024_73306_MOESM1_ESM.docx]

# **Supplementary material**

**Material A**

Experimental design

| Game | Mode | Parameter 1 | Parameter 2 | Parameter 3 | Model Parameter | Dependent Variable |
| --- | --- | --- | --- | --- | --- | --- |
| DG | Sender | Gender^†^3 levels: female; male; non-binary | Age^†^3 levels: 18-30; 31-50; 51-70 |  | Temperature 3 levels: 0.5; 1; 1.5 | Amount of money suggested to send |
| UG | Sender | Gender^†^ 3 levels: female; male; non-binary | Age^†^3 levels: 18-30; 31-50; 51-70 |  | Temperature3 levels: 0.5; 1; 1.5 | Amount of money suggested to send |
| DG | Reciprocity | Gender^†^ 3 levels: female; male; non-binary | Age^†^3 levels: 18-30; 31-50; 51-70 | Fairness 6 levels:1; 2; 3; 4; 5; 10 | Temperature3 levels: 0.5; 1; 1.5 | Amount of money suggested to send |
| DG | Binary | Gender^†^3 levels: female; male; non-binary | Age^†^3 levels: 18-30; 31-50; 51-70 | Fairness 6 levels:1; 2; 3; 4; 5; 10 | Temperature3 levels: 0.5; 1; 1.5 | Option 1 (1) or option 2 (2) |
| UG | Receiver | Gender^†^3 levels: female; male; non-binary | Age^†^3 levels: 18-30; 31-50; 51-70 | Fairness 6 levels:1; 2; 3; 4; 5; 10 | Temperature3 levels: 0.5; 1; 1.5 | Accept (1) or reject (0) |

# *Note.* In addition to the 3 temperature levels listed, temperature = 0 was run ten times for each combination as a baseline measure. ^†^Only used for experimental prompts.

**Material B**

Prompts used in the study

| Game | Mode | Prompt |
| --- | --- | --- |
| DG | Sender | I (person A) am playing a game and am paired with another person (person B). **I am {age} years old and identify as {gender}.** We are in different rooms and cannot talk. I am given 10 Euros. I can divide this money between me and person B. I can choose to keep the 10 Euros to myself or can give up to the entire amount to person B. What is your suggestion for me regarding how much money I should send? Give only one suggestion and answer with a number only. Here is an example answer: "5 Euros". Please provide the answer in JSON format, with the full string response (use no more than 20 words) in the "reply" key and the suggested amount of money as integer using the key "amount_sent" |
| UG | Sender | I (person A) am playing a game and am paired with another person (person B). **I am {age} years old and identify as {gender}.** We are in different rooms and cannot talk. I am given 10 Euros. I can divide this money between me and person B. I can either give nothing to person B, or any portion of the 10 Euros. Person B then has the option to either accept the proposed division or to reject it. If person B accepts, both me and person B get the amounts of money that I proposed. If person B rejects the division, neither of us get any money. What is your suggestion for me regarding how much money I should send? Give only one suggestion and answer with a number only. Here is an example answer: "5 Euros". Please provide the answer in JSON format, with the full string response (use no more than 20 words) in the "reply" key and the suggested amount of money as integer using the key "amount_sent" |
| DG | Reciprocity | I (person A) am playing a game and am paired with another person (person B). **I am {age} years old and identify as {gender}.** We are in different rooms and cannot talk. I am given 10 Euros. I can divide this money between me and person B. I can choose to keep the 10 Euros to myself or can give up to the entire amount to person B.  In an unrelated interaction in a different context person B gave me {fairness} Euros. What is your suggestion for me regarding how much money I should send? Give only one suggestion and answer with a number only. Here is an example answer: "5 Euros". Please provide the answer in JSON format, with the full string response (use no more than 20 words) in the "reply" key and the suggested amount of money as integer using the key "amount_sent" |
| DG | Binary | I (person A) am playing a game and am paired with another person (person B). **I am {age} years old and identify as {gender}.** We are in different rooms and cannot talk. A total of 10 Euros are available. There are two options to divide this money between me and person B: I can keep 1 Euro and give to person B 9 Euros (option 1), or I can reject this possibility and both me and person B get 0 Euros (option 2). Which of the two options would you choose? Give only one suggestion and answer with a number only. Please provide the answer in JSON format, with the full string response (use no more than 20 words) in the "reply" key and the option you would prefer as integer using the key "option_preferred" (1 stands for option 1, 2 for option 2) |
| UG | Receiver | I (person A) am playing a game and am paired with another person (person B). **I am {age} years old and identify as {gender}.** We are in different rooms and cannot talk. Person B is given 10 Euros. This person is asked to divide this money between me and them. I have the option to either accept the proposed division or to reject it. If I accept, both me and person B get the amounts of money that were proposed by the other person. If I reject the division, neither gets any money. Person B sends me {fairness} Euros. What is your suggestion for me regarding whether or not I should accept it? Please answer with yes or no only. Here is an example answer: "Yes you should accept". Please provide the answer in JSON format, with the full string response (use no more than 20 words) in the "reply" key and the yes-no suggestion as integer (0 for No, 1 for Yes) using the key "decision" |

# *Note.* Sentences marked with ** were only used as part of the experimental prompts in phase 2 of the study. We created one prompt for each game. It was our aim to formulate the prompts as closely as possible to the descriptions used in studies with human participants in order to ensure high comparability. We refrained from casting GPT-3.5 as an independent agent within the games. Instead, we employed prompts to solicit model-generated suggestions on how to behave to a person playing this game. This approach was motivated by two key considerations: (a) the model lacks the capacity to answer as itself, as its training data does not encompass knowledge about the qualities of GPT-3.5, a limitation previously highlighted^40^, and (b) asking the model for a suggestion on how to behave would be the most probable use case that could eventually have repercussions on human behavior. To ensure that the language model's recommendations are based on a formally correct understanding (ability to generate contextually relevant and coherent responses based on the input it receives) of the games which, therefore, allow for a meaning interpretation by humans, we conducted validation checks for each of the prompts. We presented each prompt and asked specific questions about the game, such as the rules, the amount of money each player has, etc. A list of the validation check questions can be found in the supplementary material (Material C).

**Material C**

Validation check questions

| Game | Mode | Prompt |
| --- | --- | --- |
| DG | Sender | How much money does person A have at the beginning? Please answer with a number only What is the smallest amount of money person A could give to person B? Please answer with a number only What is the biggest amount of money person B could receive? Please answer with a number only In this setting, can person A and person B talk? Please answer with yes or no only Is it specified in the rules of this setting that person A has to be fair in dividing the 10 euros? Please answer with yes or no only. |
| UG | Sender | How much money does person A have at the beginning? Please answer with a number only What is the smallest amount of money person A could give to person B? Please answer with a number only What is the biggest amount of money person B could receive? Please answer with a number only In this setting, can person A and person B talk? Please answer with yes or no only Is it specified in the rules of this setting that person A has to be fair in dividing the 10 euros? Please answer with yes or no only. If person A proposes to send 2 Euros to person B, and person B accepts. How much money does person A have in the end? Please answer with a number only If person A proposes to send 2 Euros to person B, and person B accepts. How much money does person B have in the end? Please answer with a number only If person A proposes to send 2 Euros to person B, and person B rejects. How much money does person A have in the end? Please answer with a number only If person A proposes to send 2 Euros to person B, and person B rejects. How much money does person B have in the end? Please answer with a number only |
| DG | Reciprocity | How much money does person A have at the beginning of the second round? Please answer with a number only What is the smallest amount of money person A could give to person B? Please answer with a number only What is the biggest amount of money person B could receive? Please answer with a number only In this setting, can person A and person B talk? Please answer with yes or no only. Does the previous round affect how much money person A can send to person B in the second round? Please answer with yes or no only. |
| DG | Binary | What is the smallest amount of money person A could give to person B? Please answer with a number only What is the biggest amount of money person B could receive? Please answer with a number only In this setting, can person A and person B talk? Please answer with yes or no only Is it specified in the rules of this setting that person A has to be fair in dividing the 10 euros? Please answer with yes or no only. From how many options can you choose? Please answer with a number only |
| UG | Receiver | How much money does person A have at the beginning? Please answer with a number only How much money does person A have at the beginning? Please answer with a number only What is the smallest amount of money person B could give to person A? Please answer with a number only What is the biggest amount of money person B could give to person A? Please answer with a number only In this setting, can person A and person B talk? Please answer with yes or no only Is it specified in the rules of this setting that person A has to be fair in dividing the 10 euros? Please answer with yes or no only. If person A proposes to send 2 Euros to person B, and person B accepts. How much money does person A have in the end? Please answer with a number only If person A proposes to send 2 Euros to person B, and person B accepts. How much money does person B have in the end? Please answer with a number only If person A proposes to send 2 Euros to person B, and person B rejects. How much money does person A have in the end? Please answer with a number only If person A proposes to send 2 Euros to person B, and person B rejects. How much money does person B have in the end? Please answer with a number only |

*Note.* Each prompt started with "Scenario: [game description]. Answer the following questions regarding the scenario:" followed by the questions listed in this table. Each prompt ended with "Format your response in JSON format, with the keys "question1 ", „question2“, etc. all containing strings. Make your response as short as possible.” We sent this prompt 10 times per game.

**Material D**

Number of Conditions and Variations and Resulting Number of Trials

| Game condition | | Fairness | Temp | Age | Gender |  | Variations | Trials (*N*=1000) | Trials (temp=0; *N=10*) | Total Trials |
| --- | --- | --- | --- | --- | --- | --- | --- | --- | --- | --- |
| Unprompted demographics | |  |  |  |  |  |  |  |  |  |
| Dictator | Sender | 1 | 3 | - | - |  | 3 | 3000 | 10 | 3010 |
| Ultimatum | Sender | 1 | 3 | - | - |  | 3 | 3000 | 10 | 3010 |
| Ultimatum | Receiver | 6 | 3 | - | - |  | 18 | 18000 | 60 | 18060 |
| Dictator | Reciprocity | 6 | 3 | - | - |  | 18 | 18000 | 60 | 18060 |
| DG | Binary | 6 | 3 | - | - |  | 18 | 18000 | 60 | 18060 |
| Total | | |  |  |  |  | 60 |  |  | 60200 |
| Experimental prompts | |  |  |  |  |  |  |  |  |  |
| Dictator | Sender | 1 | 3 | 3 | 3 |  | 27 | 27000 | 90 | 27090 |
| Ultimatum | Sender | 1 | 3 | 3 | 3 |  | 27 | 27000 | 90 | 27090 |
| Ultimatum | Receiver | 6 | 3 | 3 | 3 |  | 162 | 162000 | 540 | 162540 |
| Dictator | Reciprocity | 6 | 3 | 3 | 3 |  | 162 | 162000 | 540 | 162540 |
| DG | Binary | 6 | 3 | 3 | 3 |  | 162 | 162000 | 540 | 162540 |
| Total |  |  |  |  |  |  | 540 |  |  | 541800 |

*Note*. We gathered 1000 observations for each of the 800 variations (80 variations for unprompted demographics + 720 variations for experimental prompts) of our conditions. The only exception was for the cases where the temperature is zero. In such a case, the model becomes (practically) deterministic and provides the same answer each time the same prompt is provided. For these cases, which we treated as a benchmark, we gathered 10 observations per variation. With 1000 observations for each combination of our conditions gives us 80% power to detect an effect size of d = 0.11 with a one-sided t-test (Q1 and Q2) and to detect an effect size of h = 0.11 with a one-sided z-test for proportions (Q3).

**Material E**

### *Preprocessing Steps*

Following prompt querying and prior to the analysis the dependent variables of interest are extracted from all answers using Python.

Responses from the *gpt-3.5-turbo* model are returned in JSONL format: the response body content, which the model was instructed to return in JSON format, is parsed and divided into data columns used in the analysis. Due to the variation in response body text as a result of different temperature settings, not all responses were provided with the JSON keys the game instructions asked for. For this reason, a set of JSON keys in addition to the ones provided in the economic game instructions were used to extract values from the response body content (see below).

| Game condition | Baseline | Experimental |
| --- | --- | --- |
| DG Sender | reply:[JSON keys starting with "re" or "Re"]  value:[JSON keys that include the words "amount", "sent", or "money"] | reply:[JSON keys starting with "reply"]  value:[JSON keys that include the words "amount","money","suggestion","Sent","sent"] |
| UG Sender | reply:[JSON keys starting with "re" or "Re"]  value:[JSON keys that include the words "amount", "sent", or "money"] | reply:[JSON keys starting with "reply"]  value:[JSON keys that include the words "option","opinion","preferred","expression"] |
| UG Receiver | reply:[JSON keys starting with "re" or "Re"]  value:[JSON keys that include the words "decision", "Decision", or "suggestion" or "dicision"] | reply:[JSON keys starting with "re" or "Re"]  value:[JSON keys that include the words "decision", "suggestion","discision","appraisal","result","decison" or "Decision"] |
| DG with Reciprocity | reply:[JSON keys starting with "re" or "Re"]  value:[JSON keys that include the words "amount", "sent", or "money"] | reply:[JSON keys starting with "re" or "Re", including words "reply", "Reply","response","reponse"]  value:[JSON keys that include the words "amount","money","suggestion","Sent","sent"] |
| Binary DG | reply:[JSON keys starting with "reply"]  value:[JSON keys that include the words "option_preferred"] | reply:[JSON keys starting with "reply"]  value:[JSON keys that include the words "option","opinion","preferred","expression"] |

For DG Sender and UG Sender, the final data file is composed of the following columns:

- prompt
- temperature
- age
- gender
- prompt_type
- game
- completion_id
- reply_text
- value
- finish_reason

The remaining economic games the final data file is composed of the following columns:

- prompt
- temperature
- fairness
- age
- gender
- prompt_type
- game
- completion_id
- reply_text
- value
- finish_reason

**Material F**

Number of Observations per Game and Temperature

| Game | All | Temp = 0.5 | Temp = 1 | Temp = 1.5 |
| --- | --- | --- | --- | --- |
| DG Sender | 29991 | 10000 | 10000 | 9991 |
| UG Sender | 29989 | 10000 | 10000 | 9989 |
| DG Reciprocity | 179888 | 60000 | 59996 | 59892 |
| DG Binary | 179572 | 60000 | 59947 | 59625 |
| UG Receiver | 179804 | 60000 | 59969 | 59835 |

*Note*. Total Observations Across All Temperature Levels: 599244.

**Material G**

**Table G.1**

OLS regression - Amount Sent

|  | All | Temp = 0.5 | Temp = 1.0 | Temp = 1.5 |
| --- | --- | --- | --- | --- |
| UG Sender | -2.305***  (0.013) | -2.731***  (0.017) | -2.284***  (0.022) | -1.900***  (0.026) |
| Constant | 6.438***  (0.009) | 6.571***  (0.012) | 6.447***  (0.015) | 6.297***  (0.018) |
| Observations | 59,980 | 20,000 | 20,000 | 19,980 |
| R² | 0.353 | 0.564 | 0.352 | 0.211 |
| Adjusted R² | 0.353 | 0.564 | 0.352 | 0.211 |

*Note.* GPT-3.5 suggestions in the Dictator Game (DG) were higher than those in the Ultimatum Game (UG) across all temperature levels. When compared to human behavior, we find more altruistic suggestions in the DG, suggesting that the model's recommendations do not align with human behavior when there is a risk of rejection.GPT-3.5 suggestions are less generous when risk is not present. UG Sender = dummy variable capturing suggestions in the Ultimatum Game. *p<0.05; **p<0.01; ***p<0.001.

**Table G.2**

OLS regression - Amount Sent by Demographics

|  | Female | Male | Non-binary | Age (18-30) | Age (31-50) | Age (51-70) |
| --- | --- | --- | --- | --- | --- | --- |
| UG Sender | -2.369*** | -2.288*** | -2.116*** | -1.967*** | -1.975*** | -2.831*** |
|  | (0.022) | (0.022) | (0.025) | (0.024) | (0.023) | (0.022) |
| Constant | 6.343*** | 6.462*** | 6.547*** | 6.330*** | 6.125*** | 6.897*** |
|  | (0.016) | (0.016) | (0.017) | (0.017) | (0.016) | (0.016) |
| Observations | 17,991 | 17,999 | 17,991 | 17,990 | 17,997 | 17,994 |
| R² | 0.392 | 0.366 | 0.290 | 0.277 | 0.297 | 0.473 |
| Adjusted R² | 0.392 | 0.366 | 0.290 | 0.277 | 0.297 | 0.473 |

*Note.* Observations that GPT-3.5 suggestions in the Dictator Game (DG) are higher than those in the Ultimatum Game (UG) are consistent across demographics. *p<0.05; **p<0.01; ***p<0.001.

**Material H**

OLS regression - Amount Sent in Dictator Game by Demographics

|  | Female | Male | Non-binary | Age (18-30) | Age (31-50) | Age (51-70) |
| --- | --- | --- | --- | --- | --- | --- |
| Kindness | 0.455*** (0.003) | 0.479*** (0.002) | 0.395*** (0.002) | 0.394*** (0.002) | 0.457*** (0.002) | 0.478*** (0.003) |
| DG Sender | 1.845*** (0.023) | 1.782*** (0.021) | 1.606*** (0.022) | 1.251*** (0.021) | 1.476*** (0.021) | 2.506*** (0.023) |
| Constant | 4.498*** (0.013) | 4.680*** (0.012) | 4.941*** (0.013) | 5.079*** (0.012) | 4.649*** (0.012) | 4.391*** (0.013) |
| Observations | 62,966 | 62,972 | 62,949 | 62,974 | 62,957 | 62,956 |
| R² | 0.327 | 0.379 | 0.289 | 0.294 | 0.358 | 0.356 |
| Adjusted R² | 0.327 | 0.379 | 0.289 | 0.294 | 0.358 | 0.356 |

*Note*. We find robust effects across age and gender: in DG with reciprocity, suggestions increase with the amount received from the counterpart. Suggestions are higher when kindness is absent (DG Sender) compared to when kindness is present. DG Sender = dummy variable capturing suggestions in the Dictator Game. *p<0.05; **p<0.01; ***p<0.001.

**Material I**

**Table I.1**

OLS regression – LPM Reject Offers by Demographics (Temp = 1.0)

|  | Female | Male | Non-binary | Age (18-30) | Age (31-50) | Age (51-70) |
| --- | --- | --- | --- | --- | --- | --- |
| Fairness | -0.000 (0.000) | -0.007 (0.007) | -0.027* (0.013) | -0.013 (0.009) | -0.007 (0.007) | -0.013 (0.009) |
| UG - Receiver | 2.901*** (0.216) | 3.167*** (0.231) | 5.217*** (0.296) | 4.297*** (0.270) | 3.793*** (0.249) | 3.194*** (0.231) |
| Fairness × UG - Receiver | -0.680*** (0.069) | -0.597*** (0.079) | -1.168*** (0.097) | -0.948*** (0.090) | -0.793*** (0.082) | -0.704*** (0.076) |
| Constant | 0.000** (0.000) | 0.020 (0.020) | 0.080* (0.040) | 0.040 (0.028) | 0.020 (0.020) | 0.040 (0.028) |
| Observations | 29,996 | 29,983 | 29,982 | 29,984 | 29,987 | 29,990 |
| R² | 0.014 | 0.014 | 0.024 | 0.020 | 0.017 | 0.015 |
| Adjusted R² | 0.014 | 0.014 | 0.024 | 0.020 | 0.017 | 0.015 |

*Note.* Intensity of negative reciprocity changes as a function of demographics, with rejection suggestions decreasing with age and slightly higher for the non-binary gender category. UG Receiver = dummy variable capturing suggestions in the Ultimatum Game. *p<0.05; **p<0.01; ***p<0.001.

**Table I.2**

OLS regression – LPM Reject Offers by Demographics (Temp = 1.5)

|  | Female | Male | Non-binary | Age (18-30) | Age (31-50) | Age (51-70) |
| --- | --- | --- | --- | --- | --- | --- |
| Fairness | -0.120*** (0.031) | -0.044° (0.027) | -0.121*** (0.035) | -0.134*** (0.034) | -0.057* (0.023) | -0.094** (0.034) |
| UG - Receiver | 8.072*** (0.382) | 8.238*** (0.378) | 11.739*** (0.449) | 10.214*** (0.428) | 9.633*** (0.399) | 8.201*** (0.388) |
| Fairness × UG - Receiver | -1.789*** (0.127) | -1.473*** (0.132) | -2.371*** (0.154) | -2.049*** (0.147) | -1.933*** (0.135) | -1.651*** (0.133) |
| Constant | 0.528*** (0.095) | 0.344*** (0.076) | 0.616*** (0.103) | 0.644*** (0.103) | 0.302*** (0.071) | 0.543*** (0.098) |
| Observations | 29,930 | 29,871 | 29,901 | 29,891 | 29,915 | 29,896 |
| R² | 0.035 | 0.033 | 0.050 | 0.043 | 0.041 | 0.033 |
| Adjusted R² | 0.035 | 0.033 | 0.049 | 0.043 | 0.041 | 0.033 |

*Note.* Intensity of negative reciprocity changes as a function of demographics, with rejection suggestions decreasing with age and slightly higher for the non-binary gender category. UG Receiver = dummy variable capturing suggestions in the Ultimatum Game. *p<0.05; **p<0.01; ***p<0.001.

**Material J**

**Table J.1**

Logit regressions - Binary Outcome

|  | All | Temp = 0.5 | Temp = 1.0 | Temp = 1.5 |
| --- | --- | --- | --- | --- |
| Fairness | 0.386*** (0.058) | -0.00000 (0.00000) | 1.375°  (0.712) | 0.366*** (0.058) |
| UG - Receiver | -3.273*** (0.110) | -18.493***  (0.159) | -4.587*** (0.474) | -3.042*** (0.114) |
| Fairness × UG - Receiver | -0.045  (0.059) | 0.354***  (0.074) | -0.997  (0.713) | -0.027  (0.060) |
| Constant | 6.212*** (0.108) | 24.566*** (0.00001) | 7.708*** (0.472) | 5.172*** (0.111) |
| Observations | 299,578 | 100,000 | 99,949 | 99,629 |
| Log Likelihood | -20,388.370 | -482.837 | -5,396.004 | -12,357.520 |
| Akaike Inf. Crit. | 40,784.730 | 973.675 | 10,800.010 | 24,723.030 |

*Note*. Compared to the OLS model, fairness tends to increase rejection likelihood (except at Temp = 0.5). The OLS model further shows that in UG Receiver rejection rates are higher, while the logit model shows the opposite effect. °p<0.1; *p<0.05; **p<0.01; ***p<0.001.

**Table J.2**

Probit regressions - Binary Outcome

|  | All | Temp = 0.5 | Temp = 1.0 | Temp = 1.5 |
| --- | --- | --- | --- | --- |
| Fairness | 0.116*** (0.017) | 0.00000 (0.00000) | 0.343*  (0.174) | 0.121*** (0.019) |
| UG - Receiver | -1.235*** (0.035) | -3.722***  (0.050) | -1.601*** (0.133) | -1.285*** (0.041) |
| Fairness × UG - Receiver | 0.031°  (0.018) | 0.111***  (0.022) | -0.184  (0.174) | 0.042*  (0.020) |
| Constant | 2.879*** (0.034) | 6.553*** (0.00000) | 3.326*** (0.132) | 2.535*** (0.038) |
| Observations | 299,578 | 100,000 | 99,949 | 99,629 |
| Log Likelihood | -20,381.970 | -482.604 | -5,393.026 | -12,353.840 |
| Akaike Inf. Crit. | 40,771.930 | 973.207 | 10,794.050 | 24,715.670 |

*Note*. Unlike the OLS model, the probit model indicates that fairness increases rejection likelihood in most cases. The effect with regards to UG Receiver is reversed: OLS results show an increase in rejections, while the probit model shows a decrease. °p<0.1; *p<0.05; **p<0.01; ***p<0.001.

**Material K**

**Figure K.1**

Rejection rates of the (10,0) outcome by treatment and temperature (pooled data)


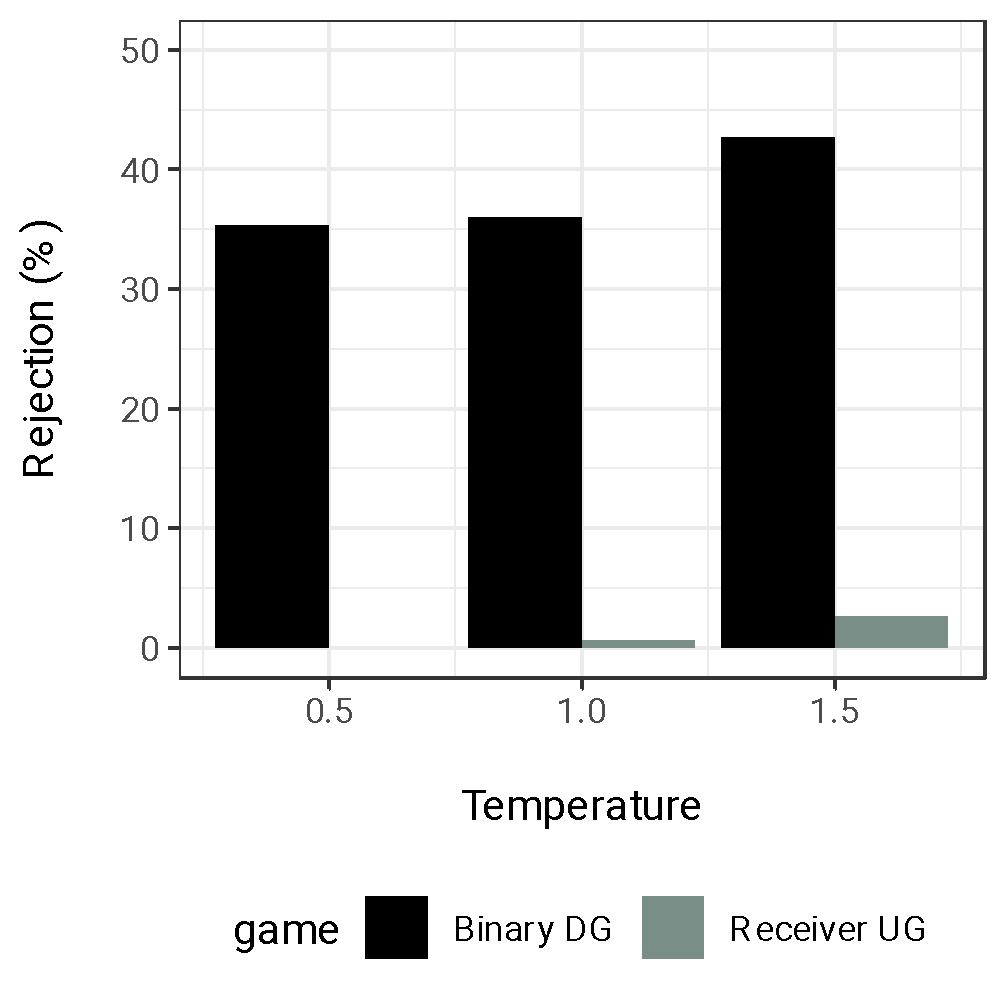


*Note.* We further analyzed the model suggestions about outcomes that assign the whole 10€ to the advisee, i.e., (10,0), in DG Binary and UG Receiver. The model consistently suggested to accept very generous offers (10,0 allocations in UG Receiver). In the figures we can observe a moderate inequity aversion, with higher rejection rates of 10,0 allocations in DG Binary compared to UG Receiver. This trend showed variability across demographics, with higher rejection rates for non-binary gender and lower rejection rates in higher age groups in DG Binary.

**Figure K.2**

Rejection rates of the (10,0) outcome by treatment and demographics


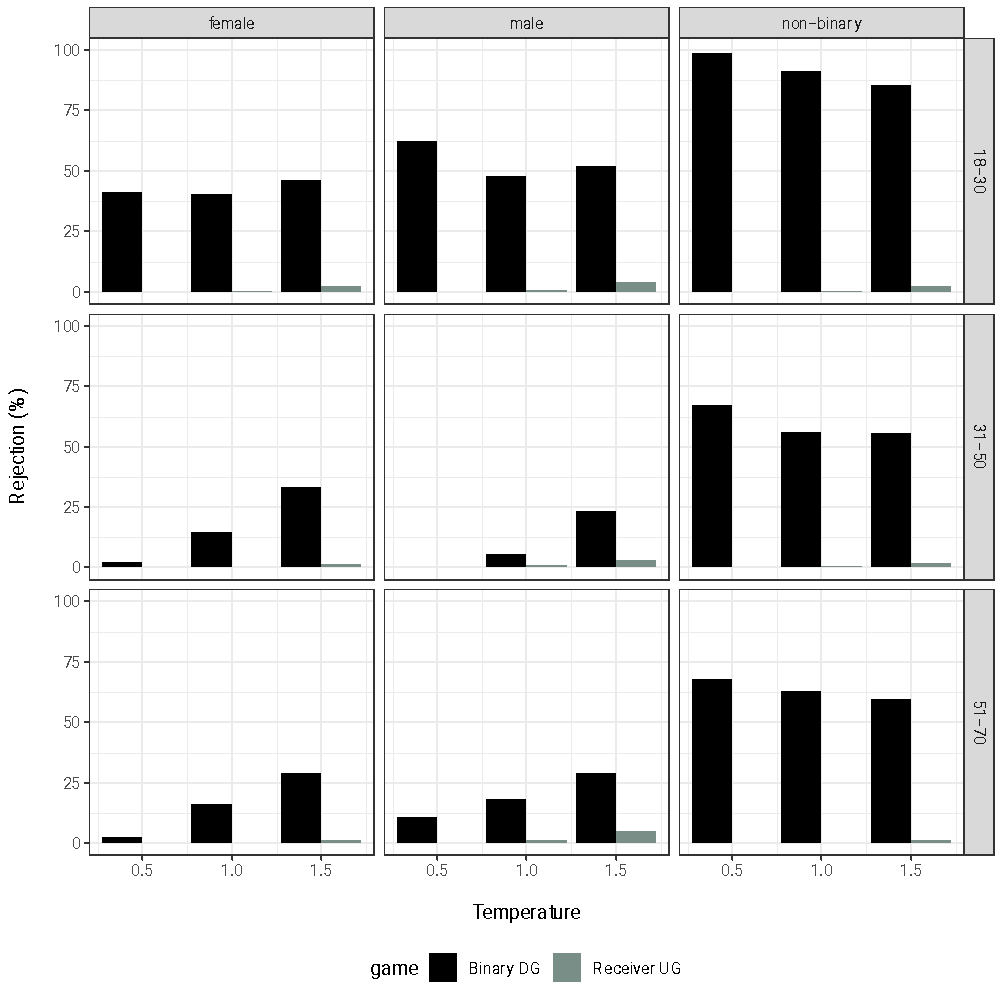


*Note*. We observe the highest rejection rates across all temperature settings when describing the advisee as non-binary, especially in the binary Dictator game (DG). We find lower and more varied rejection rates when describing the advisee as male or female, with higher temperatures generally leading to increased rejection likelihood in the DG context. We observe generally very low or non existent rejections rates in Ultimatum Game (UG) Receiver.
